# In-depth structural analysis of glycans in the gas phase

**DOI:** 10.1039/c8sc05426f

**Published:** 2019-01-04

**Authors:** Eike Mucha, Alexandra Stuckmann, Mateusz Marianski, Weston B. Struwe, Gerard Meijer, Kevin Pagel

**Affiliations:** a Fritz Haber Institute of the Max Planck Society , Department of Molecular Physics , Faradayweg 4-6 , 14195 Berlin , Germany . Email: kevin.pagel@fu-berlin.de; b Institute of Chemistry and Biochemistry , Freie Universität Berlin , Takustraße 3 , 14195 Berlin , Germany; c Oxford Glycobiology Institute , Department of Biochemistry , University of Oxford , OX1 3QU Oxford , UK

## Abstract

Although there have been substantial improvements in glycan analysis over the past decade, the lack of both high-resolution and high-throughput methods hampers progress in glycomics. This perspective article highlights the current developments of liquid chromatography, mass spectrometry, ion-mobility spectrometry and cryogenic IR spectroscopy for glycan analysis and gives a critical insight to their individual strengths and limitations. Moreover, we discuss a novel concept in which ion mobility-mass spectrometry and cryogenic IR spectroscopy is combined in a single instrument such that datasets consisting of *m*/*z*, collision cross sections and IR fingerprints can be obtained. This multidimensional data will then be compared to a comprehensive reference library of intact glycans and their fragments to accurately identify unknown glycans on a high-throughput scale with minimal sample requirements. Due to the complementarity of the obtained information, this novel approach is highly diagnostic and also suitable for the identification of larger glycans; however, the workflow and instrumentation is straightforward enough to be implemented into a user-friendly setup.

## Abstract 2

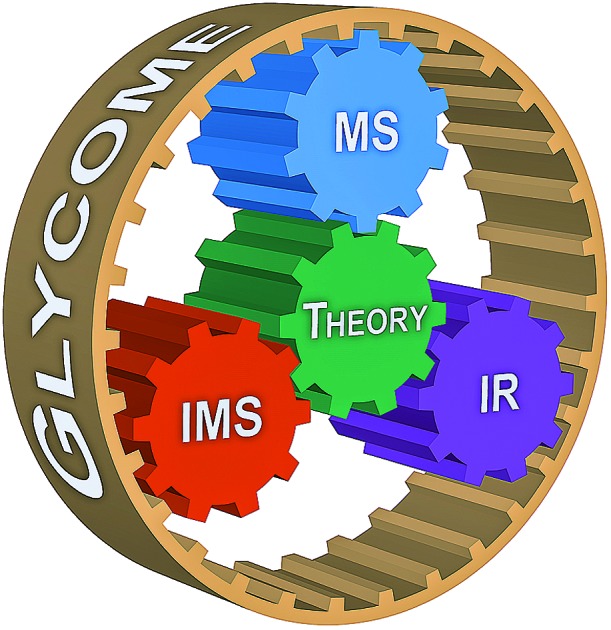
Although there have been substantial improvements in glycan analysis over the past decade, the lack of both high-resolution and high-throughput methods hampers progress in glycomics.

## Introduction

Carbohydrates-often referred to as oligosaccharides or glycans-are essential in nature, play key-roles in virtually every biological process and are altered during disease processes.^1^–^3^ The vast structural diversity enables glycans to encode rich information in biological functions including cell signaling, inflammation, immune response or molecular recognition. However, this inherent structural diversity and complex biosynthesis creates a major analytical challenge in the glycosciences and is one of the main reasons why glycomics appears to be in its infancy when compared to the rapid advances made in genomics and proteomics.^3^

The structural complexity of glycans is also reflected in their analysis, especially when compared to genomics or proteomics, which both benefit from generic sequencing methods with high-throughput and low sample consumption.^4^,^5^ To date, this remains a major hurdle in structural glycobiology. Instead, a multitude of sophisticated methods are used for glycan analysis including nuclear magnetic resonance spectroscopy^6^,^7^ (NMR), sequential mass spectrometry^8^,^9^ (MS^*n*^) or liquid chromatography^10^,^11^ (LC) as prominent examples. In this perspective article, we highlight state-of-the-art methods and present a novel concept, which combines individual techniques and optimistically provides the foundation for universal and high-throughput glycan analysis.

**Fig. 1:**
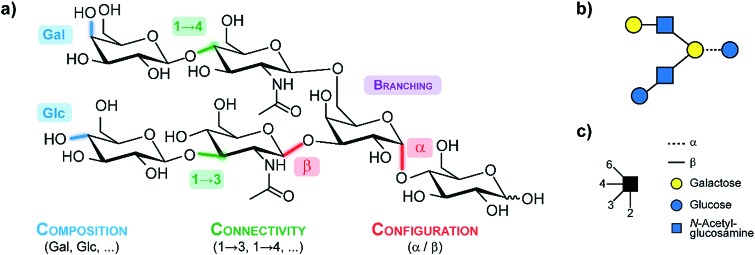
(a) A generic glycan illustrating the structural features which lead to the immense diversity in this class of biomolecules. (b) The corresponding symbol depiction according to the symbol nomenclature for glycans (SNFG)^12^ which is illustrated in (c).

An exemplary glycan is shown in (Fig. 1a), showcasing the structural features contributing to their large diversity. Monosaccharides constitute the fundamental building blocks of oligosaccharides. Larger glycans are composed of individual building blocks such as glucose, mannose or galactose, which share the same mass (composition). Two building blocks can be joined *via* glycosidic bonds in which the anomeric carbon of one sugar is linked to one of the numerous hydroxyl groups of another sugar (connectivity). In contrast to nucleic acids or amino acids, this variety of possible linkages facilities extensive branching. Furthermore, the anomeric carbon can adopt a distinct stereochemical orientation (configuration), denoted as alpha or beta. To simplify the complexity of glycan structures in a user-friendly graphical representation, the symbol nomenclature for glycans (SNFG)^12^ is typically used as shown in Fig. 1b and c.

The first part of this perspective article will provide a brief overview on the current capabilities of LC- and MS^*n*^-based methods. Generally, both methods are very powerful in their respective fields, but a detailed structural analysis often requires sophisticated sample derivatization and a time-consuming data evaluation. In the second part, we highlight ion mobility-mass spectrometry (IM-MS) as a recent addition to the structural analysis toolbox for glycans and provide a critical insight. In addition to the mass-to-charge ratio (*m*/*z*), IM-MS can separate ions according to their size and shape and provides the rotationally averaged collision cross section as an instrument independent value that is glycan structure dependent. A promising alternative for structural analysis is gas phase IR spectroscopy. Due to recent technical developments, widely tunable benchtop IR lasers (OPO/OPA) have become commercially available and are a viable alternative to IR free-electron lasers, which has led to an increased interest in the scientific community. However, classical IR multiple photon dissociation is limited to smaller glycans due to spectral broadening that arises from initial thermal contributions and the gradual heating up during the excitation process. Here, we highlight the recent developments in cryogenic IR spectroscopy as a highly sensitive method to identify glycans based on their unique and characteristic IR fingerprint.

IM-MS and cryogenic IR spectroscopy both provide detailed structural information for glycan analysis and will significantly contribute to unanswered questions in the glycosciences. However, both technologies are not universal enough and have their individual limitations, which are covered in this article. In order to fill the remaining gaps and to develop glycomics to its full potential, new analytical instruments and techniques are needed. In this context, we promote the idea of combining IM-MS and cryogenic IR spectroscopy in a single instrument to fully disentangle the structural complexity of glycans and provide a basis for a generic high-throughput method to reliably identify glycans.

Finally, we discuss the scope of theoretical methods, as we see them as compelling tools that can complement the experimental work. The proposed gas-phase experiments foster treating carbohydrates with highly accurate density-functional theory (DFT) methods instead of more common empirical force fields. We expect the theory to provide both, an atomistic basis explaining the extraordinary identification power of novel experimental techniques, and, in the future, the possibility to rigorously screen the structural space of carbohydrates to provide reference data that comprehend (or even substitute) experimental observables.

## Oligosaccharide analysis by mass spectrometry

In nature, oligosaccharides are often attached to proteins as posttranslational modifications where they play crucial roles in recognition processes and diseases. To fully unravel the biological role of such glycoproteins it is crucial to understand both, the structure of the glycan alone as well as its binding-site within the protein.^13^,^14^ Typically, the analysis of glycoproteins starts with an enzymatic digestion of the protein. Subsequently, the resulting glycopeptides are analyzed using liquid chromatography (HPLC), which for completeness will only briefly be mentioned here.

The separation in traditional reversed-phase (RP) HPLC is based on the hydrophobicity of the analyte. While the protein portion in general interacts easily with the stationary phase, glycans are hydrophilic and therefore commonly show less retention on hydrophobic stationary phases, which can result in co-eluting glycopeptides. Thus, the simultaneous analysis of the glyco – as well as the peptide-components is challenging and cannot be readily achieved. Therefore, established workflows often involve cleavage steps during which the glycan is released from the protein prior to analysis. A major drawback of this method is that site-specific glycosylation information of the intact glycopeptide is lost. The cleavage of the glycans can be achieved either chemically *via* hydrazinolysis^15^,^16^ or using enzymes such as peptide *N*-glycosidase F (PNGase F) or endoglycosidase H (endo H), which both specifically release *N*-glycans.^17^ Once hydrophilic glycans are cleaved from the protein, they can be analyzed using a multitude of chromatographic techniques including hydrophilic interaction liquid chromatography (HILIC), porous graphitic carbon chromatography (PGC), capillary electrophoresis or anion exchange chromatography. A widely used strategy to detect analytes in LC applications after separation is an ultraviolet (UV) absorption or fluorescence detection scheme. Glycans, however, are typically not UV active and therefore require sample derivatization using UV chromophores or fluorescent labels. For further reading we refer to the literature.^13^–^18^

Another increasingly important method for glycan analysis that does not require chemical derivatization is mass spectrometry (MS). Due to its sensitivity, speed, and the obtained information content, MS is one of the most widely used tools in glycan analysis.^8^,^9^,^19^ A major advantage compared to chromatographic techniques is the balance of information and effort. With MS, information about mass and consequently the composition of an oligosaccharide is obtained even for minimal amounts of sample within a short time. Depending on the chemical nature of the glycan, the analysis can be performed either in positive (as metal adduct ions) or in negative ion mode (as deprotonated ions or anion adducts), which both give rise to a different and characteristic fragmentation behavior.^20^

Among various ionization techniques, matrix assisted laser desorption/ionization (MALDI)^21^ and electrospray ionization (ESI)^22^ are the most widely used in glycan analysis. Depending on the size of the analyte, ESI yields both single and multiple charged glycans, whereas MALDI generally provides singly charged ions. In many cases, glycan isomers with an identical atomic composition and mass are present, which cannot be distinguished by MS alone and require more elaborate experiments for their reliable identification. For this purpose, tandem MS experiments are often used. Here, not only information about the glycan composition, but also connectivity and branching information can be obtained under specific conditions.^20^,^23^ The most commonly used fragmentation technique is collision induced dissociation (CID), in which selected precursor ions are subjected to collisions with neutral gas molecules to induce fragment ions. Depending on the polarity of the glycan ion, different fragmentation pathways are accessed. In positive ion mode, fragmentation mainly occurs at glycosidic bonds, *i.e.* between adjacent building blocks, which yields information about sequence and monosaccharide composition.^24^,^25^ Fragmentation of negatively charged ions on the other hand leads to cross-ring cleavages within the sugar moiety, which provides structural information about branching and the type of linkage.^20^,^26^,^27^

A general challenge in using MS and MS/MS, however, remains the unambiguous identification of isomeric species. Classes of the monosaccharide building blocks can be identified by the mass-to-charge ratio, but the identification of compositional isomers is not possible. Information on the connectivity, which is essential for the analysis of isomers, cannot be easily obtained from MS techniques without prior chemical derivatization. In addition, information about the configuration of the glycosidic bond cannot be readily obtained from these techniques alone. Another obstacle is variable response factors among glycans, with sialylated glycans exhibiting increased ionization efficiency in negative ion mode compared to equivalent neutral glycans. Furthermore, sialic acid linkages are labile which may require a derivatization step such as by permethylation^28^ of the reducing end or by methylesterification,^29^ to gather information and relative abundance of the oligosaccharide. Sequential mass spectrometry (MS^*n*^) has been shown to be very powerful in differentiating between isomers. However, data interpretation is complex and, importantly, MS^*n*^ requires considerable expertise to generate successive spectra from each glycan parent ion.^30^

One way to overcome these problems, is the use of orthogonal methods such as the combination of LC and MS. This powerful combination is currently the method of choice for the analysis of glycans^31^–^34^ as well as glycoproteins^35^,^36^ with a high reproducibility^37^ and minimal ion suppression effects.^38^ Nonetheless, there are certain limitations such as the lack of data interpretation tools that alleviate the time consuming and elaborate data analysis. Another challenge remains in the analysis of configurational and connectivity isomers. Here, often exoglycosidase^10^,^14^ are used or the sample is separated repetitively by chromatography, which increases the analysis time and sample consumption.

## Analysis of carbohydrates by ion mobility-mass spectrometry

Ion mobility spectrometry (IMS) is a powerful technique that has the ability to compensate some of the previously described problems. IMS is widespread, for example in its use in airport screening to detect explosives or chemical agents.^39^ The combination of ion mobility with mass spectrometry (IM-MS) is a promising orthogonal tool for the analysis of biomolecules.^40^–^42^ Conventional mass spectrometry detects gas-phase ions based on their mass-to-charge ratio (*m*/*z*), whereas IM separates ions according to their charge, size and shape. As a result, challenging samples such as isomers can be analyzed successfully by IM-MS, which enables new possibilities for the structural elucidation in glycomics.

**Fig. 2:**
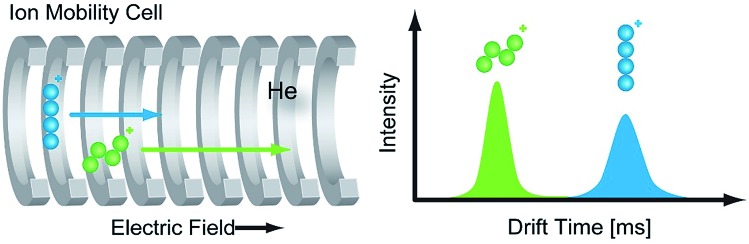
Principle of ion mobility spectrometry (IMS). In the ion mobility cell ions are separated according to their charge, size, and shape. The IM cell is filled with a neutral buffer gas such as helium or nitrogen. Gas-phase ions travel through the IM cell, guided by a weak electric field, in which larger ions (blue) undergo more collisions with the buffer gas and thus have longer drift times compared to more compact (green) ions.

In a typical IM-MS setup, ions are guided through the ion-mobility cell under the influence of a weak electric field.^43^ IM cells usually operate under constant pressure between 1–15 mbar and utilize a neutral buffer gas such as helium or nitrogen. The ions collide with the gas atoms or molecules when they traverse the cell. Due to these collisions, the ions are separated according to their mass, size, shape, and charge. Larger structures (Fig. 2, blue) collide more frequently with the buffer gas than compact structures (Fig. 2, green) and have thus longer drift times.

The experimentally-obtained drift time is an instrument-dependent value. The molecular parameter, that can be derived from the drift time is the mobility *K* from which the collision cross section (CCS) can be calculated.^44^ This rotationally-averaged area of a gas-phase ion reflects an ions' size and shape and is characteristic for each compound and thus instrument-independent. As a result, CCS values can be universally compared and allow a more accurate identification of molecules, which makes them very attractive for an implementation into databases.^45^ Currently, different types of ion mobility-mass spectrometers are commercially available.^44^

The identification and characterization of intact glycans such as *N*- and *O*-glycans by IM-MS was already demonstrated in several studies with a special focus on the differentiation between isomers.^46^–^50^ Here, an improved separation can for example be achieved by variation of the ions' charge or the investigated adduct ion.^42^,^51^–^54^ The coordination of different alkali metals as adduct ions has an effect on the conformation and CCS of the carbohydrate and as a result determines if isomers can be separated or not.^52^,^55^ Therefore, it is often necessary to investigate unknown structures in both polarities and with a variety of adduct ions to find optimal conditions for the differentiation of isomers. Another option is to increase IMS resolution, for example by applying a higher drift gas pressure^56^ or by elongating the drift cell.^57^ This, however, often requires extensive modifications of the instrument hardware, that are not always easy to implement, especially when commercial instruments are used. For future applications on complex biological samples, the development of novel IM-MS instruments with increased resolution is therefore essential.

**Fig. 3:**
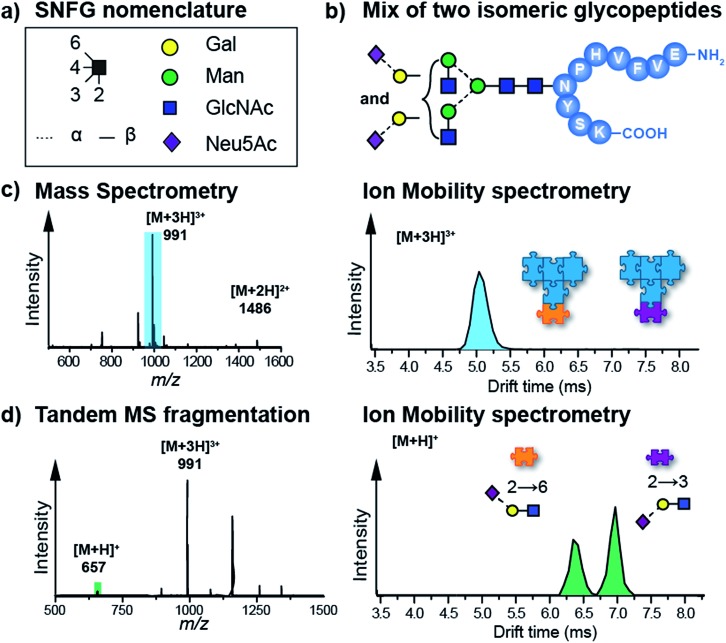
(a) Symbol nomenclature for glycan representation.^62^ (b) IM-MS analysis of an isomeric glycopeptide mixture. Both glycopeptides differ within the glycan in the connectivity of the terminal sialic acid residue (α2–6 and α2–3). (c) In a mixture, the [M + 3H]^3+^ (*m*/*z* 991) ions exhibit a single drift peak, which does not allow a separation of the two intact glycopeptides. (d) When the protonated glycopeptide precursor is fragmented prior to the IM cell, trisaccharide fragments with diagnostic drift times and CCSs are obtained.^58^,^61^

Another highly promising approach to disentangle the complex structure of carbohydrates is a fragment-based analysis using IM-MS. Especially for larger glycans, the analysis of specific fragments can be more informative than the analysis of intact isomeric precursor ions.^58^–^60^ In a recent study, isomeric glycopeptides, which only differ in the connectivity of the terminal sialic acid to the attached glycan (α2–6 or α2–3, Fig. 3b) were investigated.^58^,^61^ It is clear from the data that intact glycopeptides cannot be distinguished by means of their CCS (Fig. 3c) as only a single Gaussian shaped arrival time distribution (ATD) is observed for a mixture of both isoforms. However, CID fragmentation of glycopeptide precursor ions can yield trisaccharide B_3_ fragments carrying the terminal sialic acid residue. Depending on the linkage, these fragments show very distinct ATDs (Fig. 3d). For a mixture of both isomeric glycopeptides, the two possible B_3_ fragments are baseline-separated in IM-MS, which enables an unambiguous identification of the underlying sialic acid linkage. In a conceptually similar approach, isomeric Lewis and Blood group carbohydrate epitopes were reliably identified based on characteristic CCSs of fucosylated fragment ions.^59^

Another promising application of IM-MS is the group separation of different classes of biomolecules within a complex mixture. Due to their distinct chemical composition, different molecular classes, such as lipids, nucleotides and peptides interact differently with the drift gas. As a result, it is possible to distinguish between distinct molecular species according to the trendlines they follow in a plot of the CCS against the mass-to-charge ratio.^63^ This approach is not only of use to, for example, distinguish between peptide and glycan ions, but can also be utilized to subtract background signals arising from unwanted detergent molecules, for instance.^49^

Due to its fast duty cycle, IM-MS can be readily coupled to LC, which allows a two-dimensional analysis of carbohydrate mixtures. This method is in general very suitable for the analysis of carbohydrates, especially for the structural elucidation of isomeric compounds.^64^ However, a major challenge in this regard is the development of analysis tools that are optimized for the processing of the increasing amount of data that is obtained from these acquisitions. This is especially challenging for high-throughput applications that rely on databases. To exploit the full diagnostic capabilities of all methods, the underlying database should include information from HPLC, MS, intact glycan CCSs as well as fragment CCSs. Handling and analysis of such multidimensional datasets is in many ways demanding and will require substantial developmental effort in the future.

## Gas phase IR spectroscopy of glycans

Infrared (IR) spectroscopy is a powerful tool to investigate the vibrational modes of molecules and to deduce detailed structural information. When IR radiation is in resonance with a vibrational transition of a molecule, the absorption of photons can occur and the molecule becomes vibrationally excited. The typical fundamental vibrations are covered in the mid-IR range from 400 to 4000 cm^–1^. Classical IR spectroscopy measures the attenuation of light that is caused by the absorption of resonant photons and performs well in the condensed phase or in dense gas phase samples. However, the required large density needed for *absorption spectroscopy* cannot be fulfilled in gas-phase experiments, where the ion-density is usually limited to 10^6^ ions per cubic centimeter, due to the repulsion of equally charged ions.^65^ Instead, high-intensity laser radiation can be used to enable *action spectroscopy*. Here the absorption of photons is indirectly measured for example by following the fragmentation yield of the parent ions. The gas phase offers the unique opportunity to investigate molecules under well-defined conditions and in the absence of interactions with their environment. As such, gas phase IR action spectroscopy is already widely used to elucidate the structure of isolated small molecules, peptides or proteins.^66^ Its high sensitivity towards structural details enables the investigation concerning functional groups, intra- or intermolecular interactions and molecular conformations. But is this experimental approach useful to analyze complex carbohydrates?

The group of John P. Simons in Oxford addressed this question in the early 2000s and pioneered the early work of spectroscopic carbohydrate analysis. Following an UV-IR double resonance approach, small neutral carbohydrates were investigated in the low temperature environment of a free jet expansion.^67^–^69^ The absorption patterns obtained by UV-IR double resonance spectroscopy allowed full conformational assignments and revealed structural preferences as well as the importance of hydrogen bonding networks. However, this experimental approach is limited by the presence of a covalently bound UV-chromophore, which is usually not found in natural glycans and therefore requires a chemical derivatization. Furthermore, most species were transferred to the gas phase *via* evaporation from an oven or laser vaporization, which limits the method to smaller oligosaccharides.

A different spectroscopic approach that does not require chemical tags is infrared multiple photon dissociation (IRMPD), where the sequential absorption of multiple photons induces fragmentation of molecular ions. Measuring the fragmentation yield as a function of wavelength generates an IR spectrum. With the advance of affordable, tunable high-intensity benchtop laser systems (OPO/OPA), IRMPD spectroscopy recently emerged as a widely-used spectroscopic technique. Over the last decade, several studies explored the utility of this approach to investigate carbohydrates.^70^–^73^ Most recently, Tan *et al.* showed that lithium-adducts of *N*-acetyl-d-hexosamines reveal distinct spectral features in the hydrogen stretching region (3400–3750 cm^–1^) that can be used to distinguish these isomeric species.^73^ Moreover, theory was used to assign individual conformations and study ring-puckering, hydrogen bonding networks and the coordination site of the lithium-ion. A similar approach was followed by Schindler *et al.* elucidating the sulfation pattern of gylcosaminoglycans (GAGs) by recording IR fingerprints of individual fragments and comparing them to reference standards.^74^ In a subsequent publication, Schindler *et al.* reported that carbohydrates can retain the stereochemical information of the glycosidic bond upon fragmentation. This anomeric memory is crucial for carbohydrate sequencing and was applied to identify the sequence of chito-oligosaccharides.^75^ Recently, Martens *et al.* applied infrared ion spectroscopy to identify small metabolites from body fluid samples and observed a distinct IR signature that was assigned to *N*-acetylmannosamine (ManNAc), a biomarker for NANS-deficiency.^76^

However, a common issue in IRMPD spectroscopy is spectral congestion due to peak broadening that partly arises from the thermal activation of the ions during the sequential absorption of multiple photons.^65^ In addition, the conformational flexibility of larger oligosaccharides at room temperature may lead to the population of several coexisting conformers with different absorption patterns. The resulting spectral congestion therefore limits IRMPD spectroscopy to mono- and disaccharides.

**Fig. 4:**
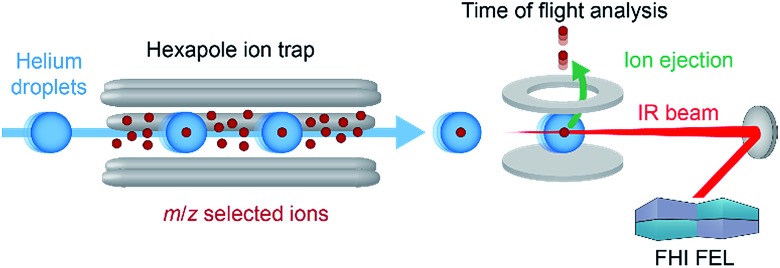
Schematic diagram of the experimental setup. Parent or fragment ions in the gas phase are mass-to-charge selected and accumulated inside an ion trap. Traversing helium droplets can pick up trapped ions, and these are immediately cooled to 0.37 K. Subsequently, the doped droplets are irradiated with IR radiation of a defined wavelength using the free-electron laser.

**Fig. 5:**
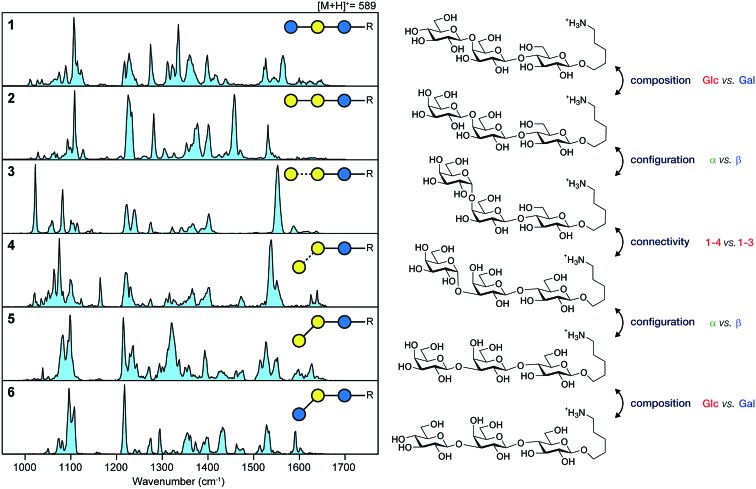
Unique IR spectra of trisaccharides 6–11, that only differ in the connectivity (1,3 or 1,4), configuration (α or β) or composition (Glc or Gal) of the terminal building block. Reproduced from ref. 77 with permission from Wiley and Sons, copyright 2017.

In a recent work, we have overcome this limitation by combining nano-electrospray ionization, mass spectrometry and ultra-cold IR spectroscopy (Fig. 4) to record well-resolved optical fingerprints of complex glycans.^77^ The unique sub-Kelvin environment of superfluid helium droplets allows to investigate isolated molecular ions in the absence of significant thermal contributions. In combination with the narrow bandwidth Fritz Haber Institute IR free-electron laser (FHI FEL^78^), spectra with an unprecedented resolution were obtained from which even minute structural variations can be resolved. A set of six isomeric trisaccharides^48^ that share an aminopentyl-linked beta-lactose core unit and only differ in the connectivity, configuration, or composition of the last building block was used to evaluate the resolving power of this experimental approach. The structures of trisaccharides 1–6, as well as their corresponding IR signatures from 950–1700 cm^–1^ are shown in Fig. 5. In the absence of significant spectral congestion, each IR signature comprises a large number of well-resolved absorption bands. The distinct spectral features allow to unambiguously distinguish between the extremely similar isomeric species and provide a unique spectral fingerprint. In addition to custom-tailored synthetic standards, the method was extended to underivatized biologically relevant samples. Two pairs of underivatized isomeric tetrasaccharides revealed unique fingerprints that confirm the high resolving power of this experimental approach and allowed to further study their fragmentation behaviour.^77^,^79^ The highly reproducible IR spectra enable the compilation of a database system that contains not only the IR signatures of intact glycan ions, but also spectra of their characteristic fragments. Especially for larger glycans, this fragment-based fingerprinting has the potential to become a key strategy for their unambiguous structural identification.

Clearly, an experimental setup that relies on superfluid helium droplets and a free-electron laser is far from a commercial application. However, there are more widely used experiments performing cold-ion spectroscopy using cold-ion traps and bench top laser systems. Usually, these experiments monitor the dissociation of a weakly bound, non-interacting messenger (or “tag”) upon irradiation to record a linear IR spectrum.^80^–^83^ As has been shown very recently, similarly diagnostic fingerprints for glycans can also be obtained using these experimental setups^84^ and even the development of user-friendly, commercial instruments is conceivable for the future.

Taken together, cold-ion spectroscopy is a valuable addition to the structural analysis toolbox for carbohydrates. Even for larger glycans, the remarkable resolving power provides a unique absorption pattern for individual isomers – a true spectral fingerprint.

## Carbohydrates as a challenge for molecular simulations

So far, we have discussed the application of emerging techniques in a phenomenological approach, without asking a fundamental question: what is the underlying structural basis that is responsible for experimental observables? To answer this, we should turn to theoretical methods. Currently, simulations of carbohydrates are largely limited to aqueous solution and employ empirical force fields to facilitate geometrical constraints imposed by condensed-phase methods such as NMR. These simulations are highly valuable when considering glycans in a biological environment, however, the methods described throughout the manuscript investigate isolated carbohydrates in a gas-phase environment. This change in the nature of the experiment bears two important consequences for theory: (1) empirical methods have not been parameterized for the gas-phase environment and (2) by peeling off solvation shells the system size is reduced. Both points advocate for reconsidering the theoretical treatment of carbohydrates and shifting from empirical force fields to more advanced quantum chemical methods, namely density-functional theory (DFT).

Carbohydrates are biopolymers like peptides or oligonucleotides and some similarities can be drawn from how those molecules are treated in simulations. The two dihedral angles of the glycosidic bond can be mapped to a Ramachandran-like representation, which similarly to the Ramachandran plot for peptides, unravels structural families that define respective arrangements of sugar rings. Such plots are unique for each type of the glycosidic bond.^85^ However, a visualization solely based on glycosidic bonds overlooks an additional degree of freedom characteristic for carbohydrates: ring puckering. Whereas peptide geometry can be approximated by a series of dihedral angle pairs connected with a rigid peptide bond, glycosidic bonds connect flexible six-membered rings, which adopt one of 38 canonical ring puckers. These puckers can be depicted according to their Cremer-Pople coordinates in a globe-like space that facilitates proximity and possible transitions between them.^86^ The space features five distinct pucker types: two chair puckers at the poles of the globe, 12 half-chairs and 12 envelopes that populate both tropics (six puckers of each type for each tropic) and six skews and six boat conformations at the equator. In most monosaccharides, steric hindrance as well as hydrogen bonding render the ^4^C_1_ chair the most stable ring pucker.

**Fig. 6:**
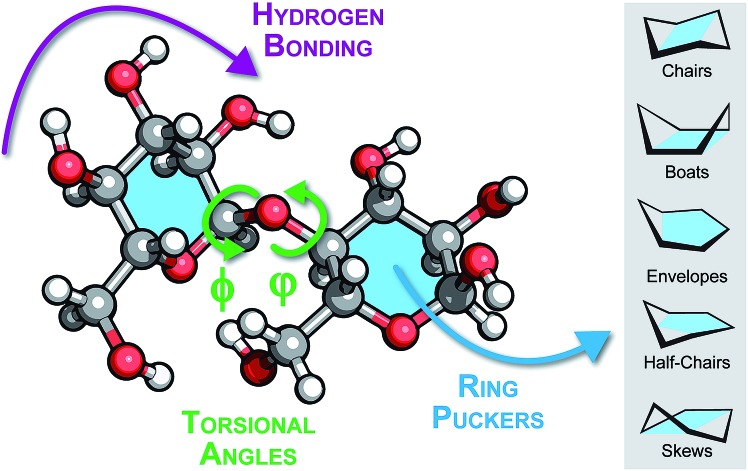
Schematic view of a disaccharide illustrating the interrelated structural features that influence the conformation of glycans: hydrogen bonding patterns or networks, the torsional angles *φ* and *φ* of the glycosidic bond and the ring puckering of individual building blocks.

In more complex oligomers, however, structural features such as hydrogen bonding, glycosidic bond geometry and rings puckering will impact the structure formation (Fig. 6). These factors are mutually coupled, for instance the interaction between rings can render non-chair puckers more stable, which in turn can drive the sugar moiety to adopt alternative glycosidic bond orientations. Subsequently, changings dihedral angles can promote hydrogen bonding patterns that stabilize distant (in sequence space) sugar branches. Understanding how these factors interplay at the atomic level should be the first step for studying structures of oligosaccharides in the gas phase. However, a fundamental question arises at this point: are empirical force fields sufficient for this task or should advanced electronic-structure theory methods be employed?

Until now, mainly empirical force fields have been widely employed for the screening of glycan conformational spaces to find low-energy conformers which would explain experimental observables. As mentioned above, and despite their success in several cases, force fields are usually limited in gas-phase studies. First, out-of-the-box force fields are generally parameterized to yield average thermodynamic quantities in bulk solution or crystals.^87^ Effectively, they are somewhat accurate in the regime they were parameterized for; they accurately assess stability of low-energy ^4^C_1_ ring puckers. However, higher-energy, less favorable non-chair ring puckers yield energetics severely underestimated by commonly employed force fields.^88^ Interestingly, even semi-empirical methods, which include electronic-structure theory contributions in their construction, behave poorly for carbohydrates. The imbalance in relative stability of chair and non-chair ring puckers can be negligible in chair-dominated aqueous solution, but it can predict incorrectly what conformations carbohydrates adopt in the gas-phase. There, a crowded glycan core can drive ring puckers towards non-chair conformers to relieve steric hindrance as well as to promote stabilizing hydrogen-bond networks. The presence of strongly interacting charges in ions introduces another disruptive force that can foster adoption of non-equilibrium conformations.^77^,^89^ Finally, the non-chair conformations can possibly modulate the slow-time dynamics of carbohydrates as a transition region between stable conformational bases. The kinetics of such transitions suffers from inadequate force fields, as shown recently exemplary for peptides.^90^

The second limitation is rooted in the force field formulation. Since empirical methods require a rigid definition of a bond connectivity for parameterization, they effectively impair bond breaking/formation processes during simulations. This is particularly challenging when studying negatively charged carbohydrates that can feature charge migration processes, as recently observed by IM-MS experiments.^48^,^89^ To describe these systems accurately, sophisticated models are required.

DFT belongs to first principles methods that do not depend on empirical parameters but are rooted in quantum mechanics. Hence, they offer out-of-the-box flexibility that makes them applicable to a wide range of systems. In principle, the same functional can be used to study protonated species, cation adducts, negative ions or molecular fragments that might arise from fragmentation processes. It neither requires an additional parameterization for heterogroups nor constrains molecular connectivity. The clean experimental gas-phase environment, moreover, reflects precisely a single-molecule calculation performed by most of the quantum chemistry packages. Finally, DFT promises a ‘chemical accuracy’ (which means that the mean absolute error is smaller than 1 kcal mol^–1^) for a diverse set of mono- and disaccharides already at the inexpensive general gradient approximation (GGA) level while popular hybrid functionals (B3LYP,^91^ PBE0 (ref. 92) or M06-2X^93^) render almost benchmark-quality results. Some novel (meta-)GGA functionals, for instance SCAN,^94^ provide an accuracy even greater than hybrid functionals at a fraction of the calculation costs. While some density functionals contain caveats in specific cases,^95^ the general use of DFT provides a fast and accurate tool for studying carbohydrates.

Nevertheless, the major obstacle in routine application of DFT to study carbohydrates becomes their vast conformational space. To save computational time, the sampling of conformational space often includes cascade-like approaches. There, the initial conformational screening is carried out using force fields and afterwards, selected structural candidates are slowly refined using increasing level of theory. This approach can be valuable but should be treated with caution: if a lower level of theory is unable to estimate a particular conformer as energetically stable and discards it, subsequent steps at higher level of theory cannot return it to the pool of candidates. To circumvent such exceptions, the search should start directly from electronic-structure calculations. Fortunately, the average carbohydrate in mammalian organisms is composed of just eight units,^96^ which can be further fragmented into smaller moieties during glycoanalysis.^59^ These entities, composed from a hundred of atoms, are tractable for routine DFT geometry optimizations, as well as calculations of infrared spectra within the harmonic approximation. Still, specific sampling approaches need to be tailored for efficient treatment of carbohydrates^88^,^97^–^100^ to reduce computational effort. However, we expect that new experimental data for oligosaccharides will foster the development of first-principles based sampling techniques.

In conclusion, density-functional theory can complement experimental data on several levels. First, it provides atomistic understanding of IM-MS and IR spectroscopic data. The development of tailored sampling techniques accesses sequences for which synthetic standards are unavailable and provides rigorous screening of the structural space in a fashion similar for short peptides. In the future, we imagine DFT will become ‘the search method’, in the meaning of the energy function that is employed in sampling algorithms. Implementation in modeling software will allow to determine the structure of isolated carbohydrates in the gas phase and either match experimental observables or replace them.

## *Quo vadis*, glycoanalysis?

Unlike for DNA and proteins, there is presently no single and universally accepted technique capable of routine, high-throughput, and comprehensive analysis of glycans. The individual methods presented above are usually dedicated towards a specific task and independently are not universal enough to provide the basis for a technological revolution in glycan analysis. Tandem mass spectrometry provides general information about the sample composition and branching (with high speed and low sample consumption) but it is intrinsically blind to the glycan's regio- and stereochemistry. Ion mobility-mass spectrometry, on the other hand, enables the separation of isomeric species or their characteristic fragments. It also determines the collision cross section, an instrument-independent value, which is directly related to the molecular conformation. Nevertheless, compositional isomers are difficult to distinguish without prior knowledge about optimal separation conditions, which renders identification of unknown samples tedious. Finally, cold-ion infrared spectroscopy provides unique fingerprints for isomeric glycans. Even minute variations present in compositional isomers are unambiguously resolved without any sample derivatization. However, the inability to preselect specific isomers from isomeric mixtures leads to a spectral congestion, which effectively conceals the identity of individual components. Therefore, to overcome shortcomings of individual techniques, we here discuss a combination of all three methods, possibly in a single instrument, as an ultimate tool to provide a basis for high-throughput analysis of glycans.

The general concept of coupling IM-MS and IR spectroscopy is already established in the research community. Among the wide range of applications, this combination can separate and identify the conformation of molecular clusters,^101^ determine secondary structure of peptides or proteins^82^,^102^–^105^ or even distinguish protomers.^106^ Coupling IM-MS and IRMPD spectroscopy, however, is still limited to the analysis of very small glycans due to peak broadening from thermal contributions, as described earlier. The combination of IM-MS and cold-ion IR spectroscopy, on the other hand, has a great potential to transform the glycosciences. The first results of combined IM-MS and cold-ion IR spectroscopy were reported by Masson *et al.*, who separated and identified two different conformers of the protonated tetrapeptide GPGG.^104^ Recently, Masellis *et al.* proved the utility of this experimental approach to distinguish a set of isomeric disaccharides and two pentasaccharides by means of their unique IR signature.^84^

**Fig. 7:**
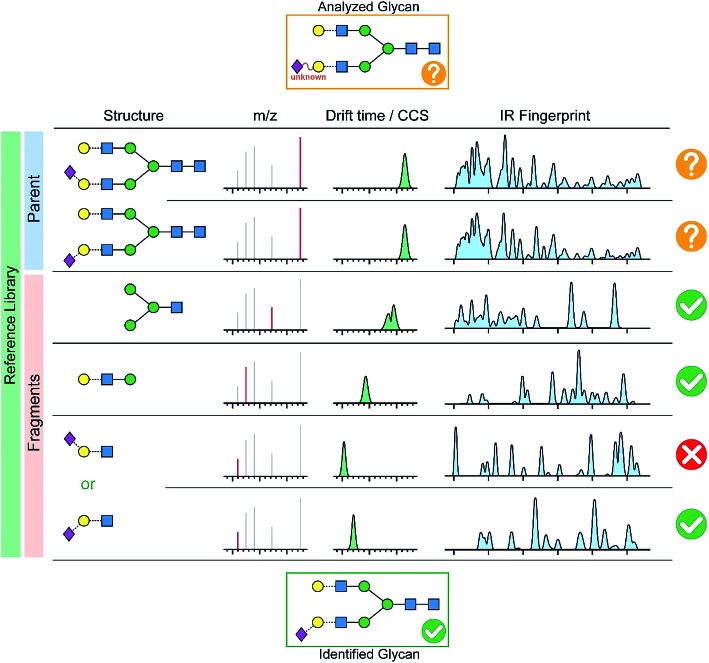
A possible database structure to aid the reliable identification of glycans including the *m*/*z* values of parent and fragment ions, their corresponding CCSs and IR fingerprints.

In order to quickly analyze a given glycan, we envision a comprehensive database in analogy to numerous LC-MS applications (*e.g.* protein analysis, pesticide residue analysis, drug determination). As sketched in Fig. 7, the database would consist of the mass-to-charge ratio, the collision cross-section and the spectroscopic fingerprint of a parent ion and its characteristic fragments. First, the fragmentation pattern, as well as the collision cross-section and the IR-fingerprint of the intact ion are compared to reference data. The definitive structure cannot be identified from this data alone due to inconclusive CCS and IR fingerprints, but we pinpoint few candidates which differ by a β-(2 → 3) and a β-(2 → 6) linked terminal *N*-acetylneuraminic acid (Neu5Ac). In this case, the analysis of characteristic fragments and a comparison to reference data becomes the key strategy. Both, ion mobility-mass spectrometry and cold-ion IR spectroscopy provide detailed complementary information for smaller fragments which unambiguously identify the analyzed glycan that contains a terminal β-(2 → 3) linked NeuAc unit.

Finally, also theory will play a crucial role in the future of glycoanalysis in several aspects. In the most fundamental approach, DFT can complement complex experimental data with detailed atomistic explanation for both collision cross section and infrared fingerprints. Effectively, such structural analysis elucidates the relation between the carbohydrate sequence and the conformation it adopts in the gas phase. However, the more immediate outcomes involve accessing the ‘no-man's-land’ of carbohydrates, *i.e.* molecules that are not readily synthetically accessible. Such unreachable carbohydrates would render empty spaces in the postulated database. Due to the very nature of theoretical methods, they can feasibly provide reference data for those missing spots. Finally, the theoretical methods can be used to rigorously screen the structural space of (smaller) fragmented carbohydrates to provide comprehensive data that covers glycospace more completely than experiments.

## Conclusions

One of the first things to learn as a young researcher in the field of glycomics is the Janus-faced nature of glycans: their immense structural diversity enables diverse biological functions making them extremely important to study, but at the same time, poses a formidable challenge in probing their structure-function relationships. Although a number of different analytical techniques are available in the glycoanalysis toolkit, the lack of efficient high-throughput, high-resolution methods for determining glycan structures greatly impedes the full development of glycomics. In this article, we first provided a brief overview on liquid chromatography and sequential mass spectrometry as basic and established tools also known in many other analytical disciplines. Although very useful, these techniques have certain limitations and leave gaps in the field of glycoanalytics. Two recently emerging and very promising techniques to bridge these gaps are ion mobility-mass spectrometry and cryogenic IR spectroscopy, both of which were critically reviewed here. However, due to individual challenges of each particular technique there is still no established single analytical instrument available to provide a generalized sequencing approach for glycans. Therefore, we suggest to compensate individual shortcomings of each method by combining IM-MS and cold-ion IR spectroscopy in a single instrument as an ultimate tool to potentially identify any glycan. The proof-of-principle of this experimental approach was recently reported using custom-build machinery. The technical development of novel ion-mobility cells and affordable benchtop laser systems leave much room for optimization and bears great potential for commercial instruments. Setting up a comprehensive reference library, which contains the *m*/*z* values, as well as collision cross-sections and IR fingerprints of intact glycans and their fragment ions may eventually enable the reliable identification of any given glycan.

## Conflicts of interest

There are no conflicts to declare.
